# The Quantum Relative Entropy of the Schwarzschild Black Hole and the Area Law

**DOI:** 10.3390/e27030266

**Published:** 2025-03-04

**Authors:** Ginestra Bianconi

**Affiliations:** School of Mathematical Sciences, Queen Mary University of London, London E1 4NS, UK; g.bianconi@qmul.ac.uk

**Keywords:** quantum gravity, entropy, black holes, Schwarzschild metric

## Abstract

The area law obeyed by the thermodynamic entropy of black holes is one of the fundamental results relating gravity to statistical mechanics. In this work, we provide a derivation of the area law for the quantum relative entropy of the Schwarzschild black hole for an arbitrary Schwarzschild radius. The quantum relative entropy between the metric of the manifold and the metric induced by the geometry and the matter field has been proposed in G. Bianconi as the action for entropic quantum gravity leading to modified Einstein equations. The quantum relative entropy generalizes Araki’s entropy and treats the metrics between zero-forms, one-forms, and two-forms as quantum operators. Although the Schwarzschild metric is not an exact solution of the modified Einstein equations of the entropic quantum gravity, it is an approximate solution valid in the low-coupling, small-curvature limit. Here, we show that the quantum relative entropy associated to the Schwarzschild metric obeys the area law for a large Schwarzschild radius. We provide a full statistical mechanics interpretation of the results.

## 1. Introduction

The area law satisfied by the thermodynamic entropy of black holes is one of the cornerstones of quantum gravity [1,2,3]. The discovery that the entropy, notoriously an extensive quantity, can obey an area law came as a big surprise of the early findings of Bekenstein [4,5] and Hawking [6,7] and continues to stimulate theoretical physics explanations. Indeed, after the discovery of this law, the study of the entropy of black holes [8,9] and the area law became a testbench for quantum gravity approaches leading to explanations making use of string theory [10], the holographic principle [11,12], the AdS/CFT correspondence [13], and in particular the Ryu–Takayanagi formula [14] and loop quantum gravity approaches [15]. The area law is also considered a universal property of condensed matter systems [16,17] as it has an important interpretation in terms of the entanglement entropy. Interestingly, recent approaches have provided new insights into black-hole entropy by quantifying the entanglement entropy of scalar fields near the horizon of black-holes [18,19].

In this work, we discuss the quantum relative entropy of the Schwarzschild black hole. The quantum relative entropy is a fundamental information theory quantity [20] whose importance is central in quantum information and the theory of quantum operators [21,22,23,24]. Recently, in Ref. [25], the quantum relative entropy has been proposed by G. Bianconi as the fundamental information theory action for the entropic quantum gravity approach. The definition of the quantum relative entropy relies on the treatment of the spacetime metric and the metric induced by the geometry of spacetime and the matter fields as quantum operators. Note that the idea that the considered manifold is described by two metrics is at the foundation of the bi-metric gravitation [26,27] as well. However, the treatment of these two metrics as quantum operators and the use of the quantum relative entropy between the two metrics as the action for gravitation make the entropic quantum gravity approach significant distinct from the bi-metric approach. In the entropic quantum gravity approach, the action for gravity is the quantum relative entropy between the metric of the considered manifold and the metric induced by the geometry and the matter field. A fundamental aspect of the entropic quantum gravity approach is that the two considered metrics are topological, i.e., they are the direct sum of metrics between zero-forms, one-forms, and two-forms. Thus, this aspect of the entropic quantum approach is in line with growing interest in area metrics in quantum gravity [28,29,30,31].

The entropic quantum gravity approach leads to modified Einstein equations, which reduce to the Einstein equations in the low-coupling, small-curvature limit. However, the action of the entropic quantum gravity is very different from the Einstein–Hilbert action. Among the important differences, we observe that, thanks to the inclusion of metrics between two-forms, the entropic quantum gravity action depends explicitly on the Riemann tensor; therefore, it is not vanishing for a Schwarzschild black hole.

In this work, we perform a derivation of the area law for the quantum relative entropy associated to the Schwarzschild black hole. It is to be noted that the Schwarzschild metric is not an exact black-hole solution of the entropic quantum gravity approach; however, it is a solution in the low-coupling, small-curvature regime. The area law is recovered exactly in this limit, i.e., when the Schwarzschild radius is very large, although the multiplicative constants are different than the ones predicted for the thermodynamic entropy. Moreover, for a small radius, deviations from the area law are observed.

Recently, we have entered a phase of experimental tests of gravity combining results coming from different experimental sources. This includes, of course, the validation coming from gravitational wave experiments [32,33] and also includes validations of analogue gravity [34,35,36] and the exploration of gravity effects by the means of quantum information theory [37,38,39,40]. Thus, it is our hope that these results can contribute to providing experimental probes of the quantum gravity effects in nature.

## 2. Boltzmann Legacy and the Quantum Relative Entropy for Gravity

Our starting point is the celebrated expression for the entropy given by Boltzmann [41], which provides a microscopic interpretation of the thermodynamics entropy S=S(E,V) for a system of given total energy *E* and volume *V*, i.e.,

(1) $$\begin{array}{c} S\left(E,V\right)=k_{B}\ln W \end{array}$$

Here, $k_{B}$ indicates the Boltzmann constant, while *W* indicates the number of microstate configurations compatible with the considered macrostate configuration. One classical result of this formula is that the entropy is extensive. This implies that for a system of locally interacting set of *N* identical particles in thermal equilibrium, which can be considered as the sum of two systems at the same temperature (a system of $N_{1}$ and a system of $N_{2}$ particles), the total number of particles *N*, the total volume *V*, and the total energy *E* of the system can be written as

(2) $$\begin{array}{c} N=N_{1}+N_{2},\\ V=V_{1}+V_{2},\\ E=E_{1}+E_{2}, \end{array}$$

where $V_{i}$ and $E_{i}$ for i∈{1,2} are the volume and the energy of the two subsystems, respectively. In this scenario, we have that *W* obeys

(3) $$\begin{array}{c} \ln W=\ln W_{1}+\ln W_{2}+O\left(\ln N\right), \end{array}$$

where $W_{i}$ is the number of microscopic configurations compatible with the subsystem *i*. It follows that the entropy S(E,V) is extensive, i.e.,

(4) $$\begin{array}{c} S\left(E,V\right)=S_{1}\left(E_{1},V_{1}\right)+S_{2}\left(E_{2},V_{2}\right)+O\left(\ln N\right). \end{array}$$

Thus, considering a system as composed by *n* macroscopic subsystems i∈{1,2,…,n} , we obtain

(5) $$\begin{array}{c} S\left(E,V\right)=k_{B}\sum_{i=1}^{n}\ln W_{i}. \end{array}$$

In gravity, the degrees of freedom are encoded in the spacetime fabric of a d=4 dimensional manifold K of Lorentzian signature {−1,1,1,1} , whose geometry is fully described by its metric $g_{\mu \nu }$ . In the entropic quantum gravity proposed in Ref. [25], the topological metric considered comprises the metric among scalars, the metric among vectors, and the metric among bivectors defined in K . This is given by

(6) $$\begin{array}{ccc} \tilde{g} & = & 1⊕g_{\mu \nu }dx^{\nu }⊗dx^{\nu }\\ & & ⊕{\left[g_{\left(2\right)}\right]}_{\mu \nu \rho \sigma }\left(dx^{\mu }\wedge dx^{\nu }\right)⊗\left(dx^{\rho }\wedge dx^{\sigma }\right). \end{array}$$

where

(7) $$\begin{array}{c} {\left[g_{\left(2\right)}\right]}_{\mu \nu \rho \sigma }=\frac{1}{2}\left(g_{\mu \rho }g_{\nu \sigma }-g_{\mu \sigma }g_{\nu \rho }\right). \end{array}$$

Additionally the topological metric $\tilde{G}$ induced by the geometry and the matter fields is also considered; this metric also comprises the direct sum between a metric among scalars ${\tilde{G}}_{\left(0\right)}$ , a metric among vectors ${\tilde{G}}_{\left(0\right)}$ , and a metric among bivectors ${\tilde{G}}_{\left(1\right)}$ and is given by

(8) $$\begin{array}{ccc} \tilde{G} & = & {\tilde{G}}_{\left(0\right)}⊕{\left[{\tilde{G}}_{\left(1\right)}\right]}_{\mu \nu }dx^{\mu }⊗dx^{\nu }\\ & & ⊕{\left[{\tilde{G}}_{\left(2\right)}\right]}_{\mu \nu \rho \sigma }\left(dx^{\mu }\wedge dx^{\nu }\right)⊗\left(dx^{\rho }\wedge dx^{\sigma }\right), \end{array}$$

where at each point *p* of the manifold K , the matrices ${\tilde{G}}_{\left(m\right)}$ with m∈{0,1,2} are invertible. The dual metric is given by ${\tilde{G}}^{★}$

(9) $$\begin{array}{ccc} {\tilde{G}}^{★} & = & {\tilde{G}}_{\left(0\right)}⊕{\left[{\tilde{G}}_{\left(1\right)}\right]}^{\mu \nu }dx_{\mu }⊗dx_{\nu }\\ & & ⊕{\left[{\tilde{G}}_{\left(2\right)}\right]}^{\mu \nu \rho \sigma }\left(dx_{\mu }\wedge dx_{\nu }\right)⊗\left(dx_{\rho }\wedge dx_{\sigma }\right). \end{array}$$

**Figure 1:**
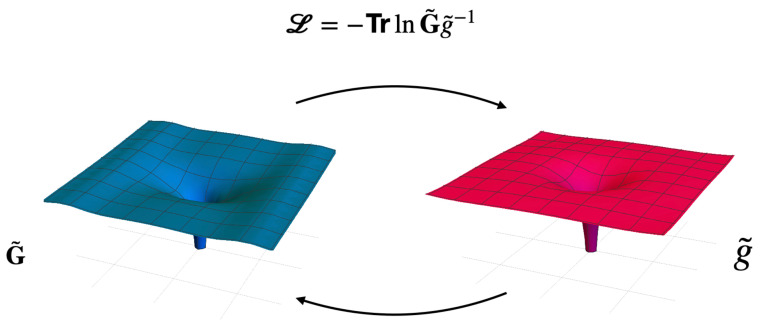
Diagrammatic sketch of the entropic quantum gravity approach. In this approach the action is given by the quantum relative entropy between the metric $\tilde{g}$ and the metric $\tilde{G}$ induced by the matter fields and the geometry of the manifold.

The entropic quantum gravity approach proposed in Ref. [25] considers the following entropic action for modified gravity given by the quantum relative entropy between $\tilde{G}$ and *g* (see Figure 1 for a diagrammatic description), i.e.,

(10) $$\begin{array}{c} S=\frac{1}{\ell_{P}^{d}}\int \sqrt{\left|-g\right|}Ldr, \end{array}$$

where $\ell_{P}={\left(\hbar G/c^{3}\right)}^{1/2}$ is the Planck length, and the Lagrangian is given by

(11) $$\begin{array}{ccc} L & := & -\mathrm{Tr}\ln\tilde{G}{\tilde{g}}^{-1}\\ & := & -\ln{\tilde{G}}_{\left(0\right)}-\mathrm{Tr}\ln{\tilde{G}}_{\left(1\right)}g^{-1}-\mathrm{Tr}\ln{\tilde{G}}_{\left(2\right)}g_{\left(2\right)}^{-1}. \end{array}$$

where here and in the following by Tr we always imply the flattened trace defined in Ref. [25] and indicated there as Tr_*F*_. Here, we assume that $\tilde{G}{\tilde{g}}^{-1}$ is positively defined, i.e., ${\tilde{G}}_{\left(0\right)}>0$ and ${\tilde{G}}_{\left(1\right)}g^{-1}$ , as well as ${\tilde{G}}_{\left(2\right)}g_{\left(2\right)}^{-1}$ , are positively defined at each point *p* of the manifold K . Note that this entropic action is expressed in terms of the square root of the modular operator $\Delta_{\tilde{G},\tilde{g}}^{1/2}$ given by

(12) $$\begin{array}{c} \tilde{G}{\tilde{g}}^{-1}=\Delta_{\tilde{G},\tilde{g}}^{1/2}=\sqrt{\tilde{G}{\tilde{G}}^{★}}, \end{array}$$

thus generalizing the definition of the Araki entropy [21] between quantum operators to the considered topological metrics $\tilde{g}$ and $\tilde{G}$ (see Ref. [25] for a more detailed discussion).

We observe that the action for the entropic quantum gravity approach also admits an information theory interpretation akin to the Boltzmann entropy. In fact, we have that the Lagrangian L can be written as

(13) $$\begin{array}{c} L=-\mathrm{Tr}\ln\tilde{G}{\tilde{g}}^{-1}=\ln W\left(r\right), \end{array}$$

where W(r) “counts” the degrees of freedom of the geometry, albeit it is in general a real rather than an integer number. In particular we have

(14) $$\begin{array}{c} W\left(r\right)={\tilde{G}}_{\left(0\right)}^{-1}\det\left({\tilde{G}}_{\left(1\right)}^{-1}g\right)\det\left({\tilde{G}}_{\left(2\right)}^{-1}g_{\left(2\right)}\right). \end{array}$$

Consequently the quantum relative entropy S can be written in a way reminiscent of Equation (5) as

(15) $$\begin{array}{c} S=\frac{1}{\ell_{P}^{4}}\int \sqrt{-|g|}\ln W\left(r\right)dr. \end{array}$$

Thus, the quantum relative entropy counts the number of degrees of freedom of the metric and is associated with the volume over which the integral is performed.

## 3. Modified Einstein Equations in Vacuum

The entropic quantum gravity approach leads to modified Einstein equations, which reduce to the Einstein equation in a regime of low coupling (small curvature and low energies). Here, we are interested in discussing the corresponding modified Einstein equations in vacuum and showing that the Schwarzschild solutions are approximate solutions of these modified Einstein equations in the low-coupling regime. In vacuum, adopting the units ℏ=c=1 , the expression of the metric induced by the geometry is assumed (see Ref. [25]) to be given by

(16) $$\begin{array}{c} \tilde{G}=\tilde{g}-\beta G\tilde{R}, \end{array}$$

where *G* is the gravitational constant, *β* is a adimensional constant and $\tilde{R}$ is given by the topological curvature, comprising the Ricci scalar *R*, the Ricci tensor $R_{\mu \nu }$ , and the Riemann tensor $R_{\mu \nu \rho \sigma }$ , i.e.,

(17) $$\begin{array}{ccc} \tilde{R} & = & R⊕\left(R_{\mu \nu }dx^{\mu }⊗dx^{\nu }\right)\\ & & ⊕R_{\mu \nu \rho \sigma }\left(dx^{\mu }\wedge dx^{\nu }\right)⊗\left(dx^{\rho }\wedge dx^{\sigma }\right). \end{array}$$

Leaving the discussion of the derivation of the modified Einstein equations derived from the entropic quantum gravity action S to Ref. [25], here, we summarize their structure. The modified Einstein equations of entropic quantum gravity involve two sets of equations: the equations for the metric $\tilde{g}$ and the equations for the auxiliary G-fields $\tilde{G}$ (a form of auxiliary metric as well).

The equations for the G-fields $\tilde{G}$ are given by

(18) $$\begin{array}{c} {\tilde{G}}^{-1}=\tilde{I}-\beta G\tilde{R}{\tilde{g}}^{-1}, \end{array}$$

where ${\tilde{G}}^{-1}$ is the topological metric comprising a metric among scalars, one among vectors, and one among bivectors, each equal to the inverse of the corresponding metrics forming the topological metric $\tilde{G}$ , and $\tilde{I}$ is the topological identity metric.

The modified Einstein equations for the metric $\tilde{g}$ are given by

(19) $$\begin{array}{c} R_{\left(\mu \nu \right)}^{G}-\frac{1}{2}g_{\mu \nu }\left(R_{G}-2\Lambda_{G}\right)+D_{\left(\mu \nu \right)}=0, \end{array}$$

where

(20) $$\begin{array}{ccc} R_{G} & = & {\mathrm{Tr}}_{F}{\tilde{g}}_{G}^{-1}\tilde{R},\\ \Lambda_{G} & = & \frac{1}{2\beta G}{\mathrm{Tr}}_{F}\left(\tilde{G}-\tilde{I}-\ln\tilde{G}\right), \end{array}$$

with ${\tilde{g}}_{G}$ indicating a “dressed metric” given by

(21) $$\begin{array}{c} {\tilde{g}}_{G}={\tilde{G}}^{-1}g. \end{array}$$

Note that in Equation (19), (μν) indicates the symmetrization of the indices, $R_{\mu \nu }^{G}$ are the elements or the *dressed Ricci tensor* given by

(22) $$\begin{array}{ccc} R_{\mu \nu }^{G} & = & G_{\left(0\right)}R_{\mu \nu }+{\left[G_{\left(1\right)}\right]}_{\mu }^{\;\rho }R_{\rho \nu }-{\left[G_{\left(2\right)}\right]}_{\rho_{1}\rho_{2}\mu \eta }R_{\nu }^{\;\eta \rho_{1}\rho_{2}}\\ & & +2{\left[G_{\left(2\right)}\right]}_{\mu }^{\;\eta \rho_{1}\rho_{2}}R_{\rho_{1}\rho_{2}\nu \eta }, \end{array}$$

while $D_{\mu \nu }$ are the elements depending on second derivatives of the G-field $\tilde{G}$ given by

(23) $$\begin{array}{ccc} D_{\mu \nu } & = & \left(\nabla^{\rho }\nabla_{\rho }g_{\mu \nu }-\nabla_{\mu }\nabla_{\nu }\right)G_{\left(0\right)}-\nabla^{\rho }\nabla_{\nu }{\left[G_{\left(1\right)}\right]}_{\left(\rho \mu \right)}\\ & & +\frac{1}{2}\nabla^{\rho }\nabla_{\rho }{\left[G_{\left(1\right)}\right]}_{\mu \nu }+\frac{1}{2}\nabla^{\rho }\nabla^{\eta }{\left[G_{\left(1\right)}\right]}_{\rho \eta }g_{\mu \nu }\\ & & +\nabla^{\eta }\nabla^{\rho }{\left[G_{\left(2\right)}\right]}_{\mu \rho \nu \eta }+\nabla^{\rho }\nabla^{\eta }{\left[G_{\left(2\right)}\right]}_{\eta \mu \rho \nu }\\ & & +\frac{1}{2}\left[\nabla^{\rho },\nabla^{\eta }\right]{\left[G_{\left(2\right)}\right]}_{\rho \eta \mu \nu }. \end{array}$$

These modified Einstein equations reduce to the Einstein equations in vacuum

(24) $$\begin{array}{c} R_{\mu \nu }=0, \end{array}$$

only if

(25) $$\begin{array}{c} {\tilde{G}}^{-1}=\tilde{I}, \end{array}$$

i.e., only if

(26) $$\begin{array}{c} \tilde{R}{\tilde{g}}^{-1}=0. \end{array}$$

However, the Einstein equations in vacuum remain a good approximation of the modified Einstein equations as long as

(27) $$\begin{array}{c} \beta G\tilde{R}{\tilde{g}}^{-1}\ll \tilde{I}. \end{array}$$

Thus, the Schwarzschild metric can be interpreted only as an approximate solution of the modified Einstein equations of entropic quantum gravity in vacuum valid in the regime of small curvature. This implies that if the entropic quantum gravity approach captures the physics of gravitation, the physical black holes will only be described by the Schwarzschild metric in a linear approximation valid in the small-curvature regime.

Relevantly, however, we observe that the quantum relative entropy S between $\tilde{G}$ and $\tilde{g}$ , given by Equation (16) is defined for any metric, not only for the metric satisfying the mentioned equations for modified gravity. Thus, in the next section, we address the challenge of evaluating the quantum relative entropy S of the Schwarzschild metric.

## 4. Quantum Relative Entropy of the Schwarzschild Black Hole

In this section, our goal is to provide the derivation of the quantum relative entropy of the Schwarzschild black hole. In particular, we show that the quantum relative entropy of the Schwarzschild black hole follows an area law for large Schwarzschild radii. The starting point is the observation that the quantum relative entropy defining the entropic quantum gravity approach is not vanishing for a Schwarzschild black hole as it depends explicitly on the Riemann tensor and not just exclusively on the Ricci scalar and the Ricci tensor. This allows us to directly calculate the quantum relative entropy of the Schwarzschild black hole as a function of its Schwarzschild radius.

The Schwarzschild black hole defines the static and spherically symmetric metric

(28) $$\begin{array}{c} \;ds^{2}=-\left(1-\frac{R_{s}}{r}\right)dt^{2}+{\left(1-\frac{R_{s}}{r}\right)}^{-1}dr^{2}+r^{2}d\Omega^{2}, \end{array}$$

where, in units ℏ=c=1 , $R_{s}$ defines the Schwarzschild radius given by

(29) $$\begin{array}{c} R_{s}=2GM, \end{array}$$

and where $d\Omega^{2}=d\theta^{2}+\sin^{2}\theta d\phi^{2}$ . This is the unique static and spherically symmetric metric solution to the Einstein equations

(30) $$\begin{array}{c} R_{\mu \nu }=0. \end{array}$$

As discussed in the previous section, however, this is only the approximate solution to the entropic quantum gravity equations in vacuum, valid for small curvatures.

As mentioned before, the goal here is to calculate the quantum relative entropy S defined in Equations (10) and (11) for the Schwarzschild metric defined in Equation (28). To this end, we calculate explicitly the product between the metric induced by the geometry $\tilde{G}$ in vacuum (Equation (16)) and the topological metric ${\tilde{g}}^{-1}$ , i.e.,

(31) $$\begin{array}{c} \tilde{G}{\tilde{g}}^{-1}=\tilde{I}-\beta G\tilde{R}{\tilde{g}}^{-1}. \end{array}$$

By performing this straightforward calculation, we obtain

(32) $$\begin{array}{c} \;\tilde{G}g^{-1}=1⊕\delta_{\mu }^{\nu }dx^{\mu }⊗dx_{\nu }+\Delta_{\mu \nu }^{\;\;\rho \sigma }dx^{\mu }\wedge dx^{\nu }⊗dx_{\rho }\wedge dx_{\sigma }, \end{array}$$

where

(33) $$\begin{array}{c} \Delta_{\mu \nu }^{\;\;\rho \sigma }=\frac{1}{2}(\delta_{\mu }^{\rho }\delta_{\nu }^{\sigma }-\delta_{\mu }^{\sigma }\delta_{\nu }^{\rho })-\beta GR_{\mu \nu }^{\;\;\rho \sigma }. \end{array}$$

We then calculate the non-zero elements of the Riemann tensor $R_{\mu \nu }^{\;\;\rho \sigma }$ associated to the Schwarzschild metric to be

(34) $$\begin{array}{ccc} R_{tr}^{\;\;tr} & = & R_{\theta \phi }^{\;\;\theta \phi }=\frac{R_{s}}{r^{3}},\\ R_{t\theta }^{\;\;t\theta } & = & R_{t\phi }^{\;\;t\phi }=R_{r\theta }^{\;\;r\theta }=R_{r\phi }^{\;\;r\phi }=-\frac{R_{s}}{2r^{3}}. \end{array}$$

Inserting these expressions in the Lagrangian L defined in Equation (11), we obtain

(35) $$\begin{array}{c} \;L=-\mathrm{Tr}\ln\Delta =-\ln\left[{\left(1-2\beta \frac{GR_{s}}{r^{3}}\right)}^{2}{\left(1+\beta \frac{GR_{s}}{r^{3}}\right)}^{4}\right], \end{array}$$

where the trace of the logarithm of an area metric is defined as the trace of the matrix resulting from the flattened tensor as discussed extensively in Ref. [25]. Here the Lagrangian L is defined as long as Δ is positively defined, i.e., for

(36) $$\begin{array}{c} r>r_{0}={\left(2\beta GR_{s}\right)}^{1/3}={\left(4\beta G^{2}M\right)}^{1/3}. \end{array}$$

We define the entropy of the Schwarzschild black hole as

$$\begin{array}{ccc} S(R_{s},\tau ) & = & -\frac{1}{\ell_{P}^{4}}\int_{0}^{\tau }dt\int_{r_{0}}^{R_{s}}dr\int d\Omega \sqrt{-|g|}\mathrm{Tr}\ln\tilde{G}{\tilde{g}}^{-1}\\ & = & -\frac{1}{\ell_{P}^{4}}\int_{0}^{\tau }dt\int_{r_{0}}^{R_{s}}dr\int d\Omega \sqrt{-|g|}\mathrm{Tr}\ln\Delta . \end{array}$$

Since the integral has the lower bound $r_{0}$ , if follows that this entropy is defined only for $R_{s}>r_{0}$ , which implies $R_{s}>R_{0}=\sqrt{2\beta G}$ . Moreover, we observe that when performing the integral over time, in the expression for S , we consider the dimensional scale

(37) $$\begin{array}{c} \tau =\kappa^{-1}\tau^{\prime }=4GM\tau^{\prime }, \end{array}$$

where $\kappa^{-1}=4GM$ is the surface gravity. This way, we derive the explicit expression for the quantum relative entropy of the Schwarzschild black hole as a function of its radius $R_{s}$ and of $\tau^{\prime }$ given by

(38) $$\begin{array}{ccc} S(R_{s},\tau^{\prime }) & = & \frac{32\pi M\tau^{\prime }}{3G}\left[3R_{s}^{3}\ln R_{s}^{3}+6\beta GR_{s}\ln\left(\frac{3}{2}\right)\right.\\ & & -\left(R_{s}^{3}-2\beta GR_{s}\right)\ln\left(R_{s}^{3}-2\beta GR_{s}\right)\\ & & \left.-2\left(R_{s}^{3}+\beta GR_{s}\right)\ln\left(R_{s}^{3}+\beta GR_{s}\right)\right]. \end{array}$$

In the limit $R_{s}\gg 1$ , we find that $S(R_{s},\tau^{\prime })$ is linear in the Schwarzschild radius $R_{s}$ , i.e.,

(39) $$\begin{array}{c} S\left(R_{s},\tau^{\prime }\right)=64\pi M\tau^{\prime }\beta \ln\left(\frac{3}{2}\right)R_{s}, \end{array}$$

and thus, for $\tau^{\prime }=\tau_{1}^{\prime }+\tau_{2}^{\prime }$ , we find

(40) $$\begin{array}{c} S\left(R_{s},\tau^{\prime }\right)=S\left(R_{s},\tau_{1}^{\prime }\right)+S\left(R_{s},\tau_{2}^{\prime }\right), \end{array}$$

and for $R=R_{s,1}+R_{s,2}$ with $R_{s,i}\gg 1$ ,

(41) $$\begin{array}{c} S\left(R_{s},\tau^{\prime }\right)=S\left(R_{s,1},\tau^{\prime }\right)+S\left(R_{s,2},\tau^{\prime }\right). \end{array}$$

In the above expression, we have considered $R_{s}$ as an independent variable from *M*. Let us now impose that $R_{s}=2GM$ and consider the change of variables such that the entropy becomes a function of *M* and $\tau^{\prime }$ , i.e., $S=S(M,\tau^{\prime })$ . For $R_{s}\gg 1$ , i.e., M≫1 , the black-hole entropy obeys the area law with

(42) $$\begin{array}{c} S\left(M,\tau^{\prime }\right)≃S_{A}=C\frac{A}{4G}, \end{array}$$

where the area *A* of the black hole is given by

(43) $$\begin{array}{c} A=16\pi G^{2}M^{2}, \end{array}$$

and the multiplicative constant C is given by

(44) $$\begin{array}{c} C=32\ln\left(3/2\right)\beta \tau^{\prime }≃\beta \tau^{\prime }\times 12.9749\ldots . \end{array}$$

It follows that the quantum relative entropy S retains at the same time its information theory interpretation as a quantity that evaluates the local degree of freedom of the geometry, integrated over the volume of the black hole, while it can account for the emergence of the area law of the black-hole entropy.

For $0<R_{s}-R_{0}\ll 1$ , we obtain

$$\begin{array}{c} S\left(M,\tau^{\prime }\right)≃\frac{32\pi M\beta \tau^{\prime }}{3}\left(B-4\ln\left(R_{s}/R_{0}-1\right)\right)\left(R_{s}-R_{0}\right), \end{array}$$

with B=4−4ln(9/2) . Thus, S→0 as $R\to R_{0}$ . We define the temperature of the black hole as

(45) $$\begin{array}{c} \frac{1}{T}=\frac{\partial S}{\partial M}, \end{array}$$

obtaining, in the limit M≫1 ,

(46) $$\begin{array}{c} T\to \frac{T_{H}}{C}, \end{array}$$

where $T_{H}$ is Hawking’s temperature $T_{H}^{-1}=8\pi GM$ . In the limit $R_{s}\to R_{0}=\sqrt{2\beta G}$ and $M\to \sqrt{\beta /(2G)}$ , we obtain instead

(47) $$\begin{array}{c} T≃{\left[-\frac{128\pi \beta R_{0}\tau^{\prime }}{3}\ln\left(\frac{R_{s}}{R_{0}}-1\right)\right]}^{-1}\to 0. \end{array}$$

**Figure 2:**
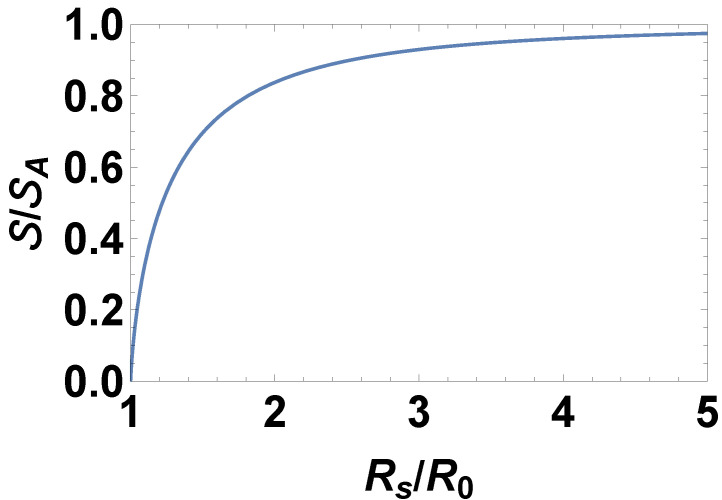
The quantum relative entropy of the Schwarzschild metric S divided by its asymptotic expression $S_{A}=CA/\left(4G\right)$ , obeying the area law, is plotted as a function of the Schwarzschild radius $R_{s}$ for G=1 .

The quantum relative entropy of the Schwarzschild metric S divided by its asymptotic expression $S_{A}$ given by Equation (42) is plotted in Figure 2 as a function of $R_{s}$ for G=1 .

We note that if entropic quantum gravity captures the true physics of gravitation, the quantum relative entropy of the Schwarzschild black hole only provides an approximation for the entropy of physical black holes, valid in the limit for large Schwarzschild radii, where the integral defining S is dominated by the terms of the small curvature. Thus, the limit $R_{s}≃\sqrt{G}$ is the one in which the entropy of the Schwarzschild metric most deviates from the entropy of the black hole described by the entropic quantum gravity equation of motion.

## 5. Conclusions

In conclusion, in this work, we considered the quantum relative entropy of a Schwarzschild black hole. The quantum relative entropy is the central action in the entropic quantum gravity proposed in Ref. [25]. It evaluates the quantum relative entropy between the metric associated with the considered manifold and the metric induced by the geometry and the matter field. This metric depends on the curvature not only through the Ricci scalar and the Ricci tensor but also through the Riemann tensor. In particular, it does not vanish for the Schwarzschild black hole that has a non-vanishing Riemann tensor. Here, we reinterpreted the Schwarzschild black-hole metric in the light of the entropic quantum gravity approach proposed in Ref. [25]. Although the Schwarzschild metric is not the exact solution of the modified Einstein equations obtained from the entropic quantum gravity approach, rather only an approximate solution, here, we calculated its associated quantum relative entropy. We showed that despite the fact that the quantum relative entropy was defined as the integral over the interior of the Schwarzschild black hole, the quantum relative entropy obeyed the area law in the limit of a large Schwarzschild radius.

This work can be expanded in several directions. On one hand, embracing the entropic quantum gravity approach will entail solving the modified Einstein equations for the corresponding black hole. On the other hand, it would be important to provide an interpretation of the quantum relative entropy in light of the second quantization of the theory. Both directions are likely to provide new quantum information insights into the entropic quantum gravity approach, which might hopefully be testable experimentally. 

## Data Availability

No data has been used or created in this work.
