# Correction: Bianconi, G. The Quantum Relative Entropy of the Schwarzschild Black Hole and the Area Law. *Entropy* 2025, *27*, 266

**DOI:** 10.3390/e27070724

**Published:** 2025-07-04

**Authors:** Ginestra Bianconi

**Affiliations:** School of Mathematical Sciences, Queen Mary University of London, London E1 4NS, UK; g.bianconi@qmul.ac.uk

## Text Corrections and Figure 2 Correction

The new version of [1] introduces corrections that quantitatively clarify the derivations in the original version of the paper and amend some typos but does not change the main message or the main results of the original paper, which remains valid and unchanged.

Different text corrections were made.

Here we list the main corrections and amendments:

(1) Reintroduction of the adimensional parameter *β* in Equation (16), carried through in subsequent equations. Since there is no emphasis in the narrative on the constant factor or the area law, this result does not change the main results of the work. However, this factor is there and can be used to numerically match the constant of proportionality of the area law with the Hawking constant. This term was in the original formulation of the theory in a previously published work in PRD [2].
(2) Corrected normalization constant typo *β* → *β*G in Equation (20). Note that in the previous version, *β* only appeared in this equation.
(3) Corrected typos regarding the topological identity in Equations (32) and (33).
(4) Corrected factor 2 calculation error in Equation (35), which was carried on in subsequent expressions. Figure 2 was changed accordingly, but the overall trend remains unchanged.

The authors state that the scientific conclusions are unaffected. This correction was approved by the Academic Editor. The original publication has also been updated.
